# The History of General Anæsthesia

**Published:** 1898-06

**Authors:** M. M. Park


### The History of General Anæsthesia.

BY M. M. PARK, D.D.S.

Read before the North-western Ohio Dental Association, April 8, 1898.

It is said "there is nothing new under the sun;" therefore it is not intended by the writer that anything new will be presented before the members of this society this evening, but rather it is his desire to revive and review that which you already know and have in part forgotten.

It will be observed that the discovery of an agent which subsequently is used for the production of anæsthesia does not enter into the discussion of the history of anæsthesia.

The subject of anæsthesia is one which received very little attention by the medical profession until within the last half century; indeed, anæsthesia as now practiced was in its infancy fifty years ago, but that a desire to free the patient from the excruciating pain of the surgeon's knife was not unknown to the surgeons of earlier ages there is abundant proof.

The use of the word "*anaesthesia*," as designating that state of unconsciousness produced by the inhalation of volatile narcotics, was first suggested by Oliver Wendell Holmes, in a letter to Dr. Morton, dated November 21, 1846, although other terms were suggested such as *nodynia*, by Sir James Y. Simpson, and *analgesia* by Prof. Roberts Bartholow, the term *anaesthesia* has become firmly fixed in usage, and is to-day repeated by the tongues of every civilized nation of mankind.

History tells us that methods of producing insensibility to pain, were in use by the ancient Greeks and Romans (who probably derived their knowledge from the Egyptians) as early as the second or third century of our era.

The agents chiefly used by them were opium, hyoscyamus and Indian hemp. Indian hemp was not only used by these nations, but by the Chinese and Hindoos about the same time. The method of use was by the inhalation of the fumes of the burning hemp.

It is thought to have been an active ingredient in the wine of the condemned, spoken of by the prophet Amos (Amos 2:8) some 700 years before the Christian era, as well as in the draught given our Saviour on the cross, to mitigate his suffering.

Another anæsthetic, mandragora, or mandrake, an indigenous plant to southern Europe, appears to have been in common use for many centuries, although for a long period prior to the discovery of modern anæsthesia it had fallen into disuse. Aetius, a Greek physician who lived about the end of the fifth century, says, those who drank of the root became sluggish, languid, dejected and cold, and fell into a heavy sleep from which it was difficult to arouse them.

Writers of the middle ages allude frequently to anæsthetic preparations; thus one writes Theodoric of Servia (P. 923 Am. S. of D.) gives receipes containing opium, the juices of green mulberry, of hyoscyamus, of conium, of leaves of mandrake, of wood ivy and the seeds of lettuce, burdock and watermelon.

There is abundant evidence that as late as the sixteenth century stupefying agents were given to criminals before being tortured, for the purpose of relieving their sufferings.

Later on, about the latter part of the eighteenth century, we we find Sir Humphrey Davy makes observations on nitrous oxide gas, who in 1800 publishes his researches concerning it and gives his experience of its power to relieve intense pain in lancing his gums to provide for the eruption of the third molar. About this time, however, his thought was directed in another channel and no more attention was given to the subject, and the knowledge of its usefulness slumbered on for nearly a half century before being brought to the attention of the world.

Other methods of producing anæsthesia have been used, such as rapid breathing for a few minutes till insensibility is attained, compression of the nerves to be operated upon, and mesmerism, upon which much has been said and written pro and con.

Thus we see that ancient, medieval and modern surgeons were anxious to welcome any agent that would abolish or mitigate the pain of surgery. Why a practical anæsthetic was not discovered before, the need being always great, is curious enough.

As we approach the fifth decade of the present century we observe that with here and there an exception, all search for anæsthetic agents had been practically abandoned, in so much that in a review of the history on anæsthesia, the distinguished French surgeon, Velpeau, about 1838, was led to use the following emphatic language: "To avoid pain under incisions is a chimera which is no longer pursued by any one. A cutting instrument and pain in surgery are two words which never present themselves separately to the mind of the patient." What a revelation was wrought in the next ten years, during which the anæsthetic property of nitrous oxide, ether and chloroform was discovered. How different the utterance of this same gentleman in 1847, when he said in a letter to a friend: "This very morning I have removed an enormous cancer from the thigh of a man who had not the slightest perception of the operation." This brings us down to the discovery of modern anæsthesia.

As was the renaissance in the sixteenth century to literature and art in Europe, so was this marvelous discovery to the science of medicine in America. What wonderful possibilities it opened up is apparent now to all, and this priceless boon was given to the world by a dentist. The discovery of our modern anæsthetics took place in rapid succession between the years of 1844 and 1847, viz.: nitrous oxide gas, ether and chloroform. Like many discoveries in other fields of science, different men laid claim to priority of discovery.

On the evening of December 10, 1844, an audience had gathered at Union Hall in the city of Hartford to witness demonstrations in chemistry by an eminent lecturer on that science (G. Q. Colton). Among other experiments an exhibition was made of the intoxicating properties of nitrous oxide gas, inhaled from a bag of oiled silk. Such exhibitions were then common, and as only stimulating doses were used, audiences were frequently amused by the grotesque and often violent antics of the subjects of the experiments. The deportment of the subjects were oftentimes so ludicrous as to cause great outbursts of laughter, thus it became known to many as *laughing gas*. At the request of some of the men after the lecture, a private exhibition was given the next day. There were present among others, Horace Wells, a practical dentist, and Samuel A. Cooley, a druggist, both of Hartford. Dr. Wells having a troublesome third molar, took the gas to have it removed, and so pleased was he with the result, that his first words after recovering from its effects were: "A new era in tooth pulling." How prophetic and true those words were we all can now attest. The practicability of the use of nitrous oxide in surgical operations, first suggested by Sir Humphrey Davy nearly fifty years before, was at last verified.

There is no evidence and very little possibility that Dr. Wells was acquainted with Davy's observation, and the honor of the first discovery and application of a safe and efficient anæsthetic agent undoubtedly belongs to him. We are proud to number him among the members of our profession. Were no other discovery made by the dental profession, this alone, is sufficient for at least a century, but another and greater anæsthetic discovery was soon to be made by another member of our noble profession. The many advances and difficult operations in surgery, by which many lives have been saved, would not have been possible were it not for it.

During the next few weeks Dr. Wells administered nitrous oxide successfully to several of his patients. That he might make his discovery known to the medical profession and also secure experiments in surgical operations other than extraction of teeth, he went to Boston in the winter of 1844–5, where he had formerly practiced dentistry. Here he called upon and obtained the assistance of Dr. Wm. T. G. Morton, a former pupil and partner of his while in that city. Dr. Morton introduced to him among others, Dr. C. I. Jackson, chemist, and Dr. J. C. Warren, professor of anatomy in Harvard University, one of the leading surgeons in New England. As no other surgical case was at once available, Dr. Warren consented to test the anæsthetic by the extraction of a tooth before his class at college.

Wells and Morton proceeded to the college, administered the gas to a patient and extracted a tooth which greatly embarrassed Wells, for the patient made considerable outcry and caused the students and spectators to laugh, and hiss, and deride him. This was probably due to some defect in the preparation of, or insufficiency of the amount of the gas administered. The chagrin and discomfiture was too much for poor Wells, and he returned home the following morning too much discouraged to make further trial of his process and in a few months he abandoned his profession and engaged in other pursuits. The visit to Boston, however, was not destined to be fruitless. No doubt it fixed the attention of Dr. Morton upon the subject and gave him the idea that surgical anæsthesia was possible.

The benefit of Dr. Wells' discovery we as dentists, and our patients, are daily reaping, as the results of his earnest endeavor. We may well bow at his shrine.

About two and a half years ago a monument was erected by the dentists of this country to his memory. It reads as follows : "This tablet commemorating the fiftieth anniversary is placed by 250 American dentists to the memory of Horace Wells, dentist, who upon this spot December 11, 1844, submitted to an operation, discovered, demonstrated and proclaimed the blessings of anæsthesia." Dr. Wells, as far as I have learned, never realized anything financially out of his discovery and after a while of brooding, sorrow and sadness, died by his own hand.

About two years after Dr. Wells' discovery, followed the discovery of anæsthesia by ether. This distinction also, we are proud to say, belongs to a dentist, the aforesaid Dr. Wm. T. G. Morton, who soon after Wells' experiment took out the degree of M. D., at Harvard; and while there, his attention was again directed to anæsthesia through the recommendation to him of chlorid ether, a solution of chloroform and alcohol as a remedy for odontalgia. In this way he came to investigate its physiological effects upon animals. The summer was spent by him in testing its effects on birds and other animals, but without satisfactory results. Morton soon then became engaged in the practice of the medical profession and gave but little more attention to the subject until 1846, when he renewed his experiments with ether and succeeded in completely anæsthetizing a dog. Thus encouraged he determined to devote his whole time to experimenting with ether and placed his practice in the hands of an assistant—anæsthesia had become the dominant thought of his life. Morton's first experiment on the human body was on himself and his two students, but the effect of the inhalation did not extend beyond the stimulant stage. An analysis proved the failure was caused by an impure article containing too much alcohol, water and acids. Morton thinking better results might be had by the use of the gas bag thought to borrow one of his preceptor, Dr. Jackson, and get if possible what information he possessed concerning the different qualities and preparations of ether. This he did in a guarded manner, fearing Jackson would forestall him in his invention should he obtain a knowledge of the full scope and purpose of his research. Dr. Jackson recommended a highly rectified article of ether to produce a partial stupefied condition, not total anæsthesia, for the knowledge that this could be produced, never possessed Dr. Jackson. This information given, however, Dr. Jackson subsequently endeavored to construe into an equal right to the discovery of the anesthetic property of ether.

Further experiments upon himself and others soon gave Dr. Morton much encouragement and he determined to bring it before the medical profession, and called upon Dr. Warren, who, undaunted by the failure of the Wells nitrous oxide experiment, gave Morton an opportunity for the practical test of his invention before the class in the Massachusetts General Hospital, October 16, 1846. The clinic room was well filled. Leading physicians of Boston came to see. At this clinic a tumor was successfully removed without pain from the side of the neck of a patient. The operation being quite dangerous owing to its relation to important nerves and blood vessels. Eager was each student and spectator to get the best vantage ground, to get a good view, expecting to hear at the first slash of the knife an unearthly scream. The operation is over in thirty minutes without a sound or struggle, at the conclusion of which Dr. Warren looked up and said: "Gentlemen, this is no humbug!" Other successful operations which soon followed in rapid succession verified the great value of this discovery to suffering humanity. News of the discovery reached England, December 17, 1846, and anæsthesia by ether soon spread to all continental Europe. Dr. Ottolengui informs us that there are still three men living who witnessed this operation. He gives the name of one only, Dr. Robert Davis, of Fall River. Up to this time the nature of the agent had not been announced, the odor of the ether being partly disguised by aromatic oils.

The medical staff of the college declined to use the preparation until informed what it was, which information was given. In this secrecy Dr. Morton felt justified until his position as discoverer was fully recognized. But Dr. Morton's troubles had just begun. Unfortunately for his fame, he endeavored to control the use of his invention in his own interest by means of letters patent which were issued him November 12, 1846, by James Buchanan, then Secretary of State. In the following month an English patent was obtained

Dr. Jackson wishing to get a piece of the pie, demanded $500 for the suggestion he had made for Morton. The matter was adjusted by accepting Jackson as a joint discoverer, giving him ten percent of the profits derived from the sale of licenses, instead of the $500. Although a few licenses were issued, the plan utterly failed, for the government failed to recognize the validity of the letters patent issued by itself.

Ether came into general use in the army and navy and throughout all the country without compensation to the discoverer. At this time others than Jackson and Morton were profuse in their claims to priority of discovery. An investigation by the French Academy of Medicine relative to the discovery of ether resulted in its giving a prize of 2,500 france to Dr. Jackson for his observations and experiments on the anaesthetic effects produced by the inhalation of ether, and the same amount to Dr. Morton for having introduced that method into surgical practice in conformity with the indications of Dr. Jackson. After much litigation in the courts the judges refused to concede to Jackson any share, direct or indirect, in the invention.

The claims of the various contestants were fully adjudicated before tribunals whose integrity can not be questioned. Their verdict must be accepted as final. Dr. Morton submitted a memorial to the 32d Congress asking remuneration for his discovery. A committee appointed by the house to review all evidence, among other conclusions announced: 1st, That Dr. Jackson does not appear at any time to have made any discovery in regard to ether, which was not in print in Great Britain some years before. "2d, That Dr. Morton in 1846 discovered the facts before unknown that ether would prevent the pain of surgical operations, and that it might be given in sufficient quantity to effect this purpose, without danger to life, first establishing the fact by numerous operations on teeth and afterward, inducing surgeons of the hospital to demonstrate its general applicability and importance in capital operations. 3rd, That the only part Dr. Jackson had in the matter was in having made suggestions which aided Dr. Morton to make the discovery, a discovery which had for some time been the object of his labors and research.

The memorial was signed by all the surgeons and physicians of the Massachusetts General Hospital and by several hundred of the leading members of the Massachusetts Medical Society. It reads as follows:

" *To the Honorable, the Senate and House of Representatives of the United States in Congress assembled:*

"'The undersigned hereby testify to your honorable bodies, that in their opinion Dr. Wm. T. G. Morton first proved to the world that ether would produce insensibility to the pain of surgical operations and that it could be used with safety. In their opinion his fellow-men owe a debt to him for this knowledge; wherefore they respectfully ask a recognition by Congress of his services to his country and mankind."

Although this memorial was sustained no grant of money was ever made to Morton, the failure to secure an appropriation being chiefly due to the antagonism not only of the friends of Jackson, but of Wells, the latter being very active in asserting his claim to the chief is not the entire merit of the discovery. Like influences caused the stoppage of a subscription in England, for Morton.

The trustees of the Massachusetts General Hospital voted him the sum of $1,000 which was presented him in a silver casket upon which was engraved this sentence: "He has become poor in a cause which has made the world his debtor." This and the 2,500 francs from the French academy was almost all Dr. Morton received for his great services to mankind.

Jenner received from the British House of Commons 30,000 pounds, equal to about $150,000, for his invention of vacination. Which has been the greatest blessing to mankind, it is difficult to say; both are priceless gifts, and it is little to the credit of America that it permitted Morton to suffer in mind, body, and estate, and to go down to his grave, to use his own words, "the only person in the world, to whom the discovery was a pecuniary loss."

Dr. Jackson after years of insanity died in 1880 in an asylum in Somerville, Massachusetts.

About one year after the discovery of anæsthesia by ether another great anaesthetic was given to the world by Sir James Y. Simpson, of Edinburgh, viz: chloroform. The anaesthetic property of this agent he discovered in 1847, and introduced the use of it into his own practice of midwifery. Since that time it has been generally used in Europe, but less in America where ether is more in use. Various combinations of different anaesthetics are in favor by some practitioners.

Regarding the relative proportion of fatal cases to the number of administrations of these anaesthetics, the following statistics are given:

| | | |
|---|---|---|
| Nitrous oxide gas (Rottenstein), | one in | 100,000 |
| Ether (Andrews), - - - - | one in | 23,204 |
| Chloroform (Lyman), - - - | one in | 5,800 |

Number of deaths recorded:

| | |
|---|---|
| Nitrous oxide, . . . . . . | 3 |
| Ether (Turnbull), 1889, . . | 52 |
| Chloroform, " " . . | 375 |

